# Higher Adherence to the AMED, DASH, and CHFP Dietary Patterns Is Associated with Better Cognition among Chinese Middle-Aged and Elderly Adults

**DOI:** 10.3390/nu15183974

**Published:** 2023-09-14

**Authors:** Ying Song, Fangxiao Cheng, Yage Du, Jie Zheng, Yu An, Yanhui Lu

**Affiliations:** 1 School of Nursing, Peking University, Beijing 100191, China; sy20000119@163.com (Y.S.); yuanfang166@163.com (Y.D.); zhengj1_27@163.com (J.Z.); 2 Institute of Medical Technology, Peking University Health Science Center, Beijing 100191, China; chengfangxiao@bjmu.edu.cn; 3 Endocrinology Department, Beijing Chaoyang Hospital, Beijing 100020, China; anyu900222@126.com

**Keywords:** alternate Mediterranean diet, dietary approaches to stop hypertension, Chinese food pagoda, cognitive function, Chinese, adults

## Abstract

The available evidence regarding the association between adherence to the Alternate Mediterranean Diet (AMED) and Dietary Approaches to Stop Hypertension (DASH) dietary patterns and cognitive performance exhibits inconsistency, and its applicability within the Asian population remains uncertain. The association between adherence to the Chinese Food Pagoda (CHFP) and cognitive function is also unknown. In this study, we aimed to assess the association between adherence to the AMED, DASH, and CHFP different dietary patterns and cognitive function. The study included 3353 Chinese adults aged 55 years and over from the China Health and Nutrition Survey (CHNS) in 2006. A 24 h dietary recall over three consecutive days was used to collect dietary information. Dietary patterns included AMED, DASH, and CHFP. A subset of items from the Telephone Interview for Cognitive Status-Modified was used for cognitive screening. Poor cognitive performance was defined as a global cognitive function score < 7. Binary logistic regression was used to estimate the association between adherence to the three different dietary patterns and cognitive function. Binary logistic regression analysis showed that there is a negative association between higher adherence to the AMED, DASH, and CHFP and poorer cognitive performance (AMED: OR = 0.594, 95% CI = 0.458–0.771, *p* < 0.001; DASH: OR = 0.652, 95% CI = 0.504–0.843, *p* = 0.001; CHFP: OR = 0.599, 95% CI = 0.417–0.861, *p* = 0.006). There was a significant interaction between each of the three dietary patterns and residential regions (AMED: *p* for interaction = 0.045; DASH: *p* for interaction = 0.003; CHFP: *p* for interaction < 0.001). Higher adherence to the AMED, DASH, and CHFP dietary patterns was inversely associated with poor cognition in Chinese middle-aged and elderly adults, particularly among urban residents.

## 1. Introduction

Cognitive impairment is a significant and growing global public health challenge in the aging population [1]. According to the latest figures from the World Health Organization, 55 million people live with dementia globally at present, with the number expected to rise to 139 million by 2050 [2]. As a form of progressive neurodegeneration, dementia severely affects the quality of life and imposes a serious economic burden [3]. The annual socioeconomic cost of dementia per patient has reached USD 19,144.36, and the national total socioeconomic cost has reached USD 167.74 billion in China alone, which is projected to rise to USD 1.89 trillion by 2050. The annual global socioeconomic cost of dementia has reached USD 957.56 billion and is projected to rise to USD 9.12 trillion by 2050 [4]. This is because there is currently no effective treatment for cognitive impairment, and the identification of potentially changeable lifestyle risk factors to prevent cognitive impairment is urgently needed.

Epidemiological studies have revealed that diets are strongly associated with cognitive impairment [5,6]. As modifiable risk factors, diets play a significant role in the prevention and management of cognitive impairment. Single foods or nutrients have a small effect on cognitive function, so the practical guidance value in relation to these is limited. Among these, dietary patterns have progressively received attention recently [7]. Dietary patterns typically encompass the combination of diverse food types and quantities consumed within one’s daily diet. This approach takes into account the interaction and synergy between components in a healthy dietary pattern and is more in line with eating behavior in real life, which provides strong justification for diet management for the prevention and control of cognitive disorders [8].

Associations between the Alternate Mediterranean Diet (AMED) and cognitive function have been observed in previous studies, providing evidence of its potential protective role. Several clinical trials have shown that adherence to the AMED is associated with improved cognitive function [9,10]. However, a clinical trial in France did not support the positive impact of Mediterranean diet adherence on cognitive function [11]. Evidence for the association between the AMED and cognitive decline is inconsistent, and data on the Asian population are limited. Likewise, a great number of investigations indicated a positive association between the Dietary Approaches to Stop Hypertension (DASH) diet and cognitive function, but the results are also inconsistent across studies. For example, in a clinical trial conducted by Smith et al., the results revealed no significant effects of the DASH intervention on neurocognitive tests, specifically pertaining to learning and memory function [12]. Furthermore, it is important to note that the aforementioned studies primarily took place in Western countries, which may exhibit dissimilarities to Asian countries due to variations in dietary habits, lifestyle factors, and other relevant aspects. The Chinese Food Pagoda (CHFP) was established following the Chinese Dietary Guidelines (CDG) issued by the Chinese Nutrition Society. Studies have largely focused on the relationship between the CHFP and cancer, cardiovascular disease, and diabetes [13]. Nonetheless, no study has examined the association between the CHFP and cognition.

In general, the findings from research investigating the association between the AMED and DASH diets and cognition exhibit incongruity, thereby raising doubts regarding their generalizability to Asian populations. To date, no research has been conducted to examine the association between the CHFP and cognitive function. Additionally, conducting an observational study that compares the associations between various dietary patterns and cognitive function could provide valuable insights into potential dietary interventions for cognition in the future. While similar studies have been conducted in the context of diseases such as colorectal cancer and diabetes, there is currently a dearth of research comparing different dietary patterns in the evaluation of cognitive function.

Therefore, the aim of this study was to examine the associations between different dietary patterns and cognitive function in Chinese adults aged 55 years and by using data from the China Health and Nutrition Survey (CHNS) to fill in this research gap.

## 2. Materials and Methods

### 2.1. Study Design and Study Population

The CHNS is the foundation of the current investigation and is an ongoing prospective cohort study that started in 1989. The National Institute for Nutrition and Food Safety, the Chinese Center for Disease Control and Prevention, and the University of North Carolina at Chapel Hill collaborated on the CHNS to investigate how Chinese social and economic transformation affects health and nutritional status [14]. The CHNS selects samples in both urban and rural areas using a multistage random cluster sampling process. The CHNS has conducted 11 waves of surveys (1989, 1991, 1993, 1997, 2000, 2004, 2006, 2009, 2011, 2015, and 2018). In the surveys performed in 1997, 2000, 2004, and 2006, participants aged 55 years and over underwent cognitive function screening tests. Further details of the study design and survey procedures have been described elsewhere [15]. The Institutional Review Board of the Chinese National Institute of Nutrition and Food Safety and the University of North Carolina approved the study. Informed consent was obtained from all participants. 

In our analysis, we used the 7th-round survey of the CHNS in 2006. The study included subjects aged ≥ 55 years who participated in cognitive function screening tests in the surveys. Subjects who did not report dietary data were excluded.

### 2.2. Measures

#### 2.2.1. Outcome Variable: Cognitive Function

A subset of items from the Telephone Interview for Cognitive Status-Modified was used for the cognitive screening; these interviews were administered by certified healthcare professionals via face-to-face interviews in the CHNS [16]. Researchers have used this tool in various population studies to evaluate cognitive function [17,18,19]. Four assignments made up the cognitive screening: (1) an immediate recall test using a list of 10 words, (2) counting backward from 20 to 1, (3) the serial 7 subtraction test, and (4) a delayed recall test using a list of 10 words. Initially, at a rate of 2 s per word, the trained health worker read out 10 words. The 10 words had to be remembered by the subjects within 2 min, and each properly remembered word received a score of 1, with the possible scores for assignment 1 ranging from 0 to 10. The subsequent task entailed the participants counting in reverse order, beginning with 20 and concluding with 1. A second chance was granted if the subjects failed the initial attempt. For the subjects who correctly counted on the first try, a score of 2 was awarded. A score of 1 was awarded to the subjects who correctly counted on the second try. Then, the subjects were required to perform 5 consecutive subtractions of 7 starting from 100. The possible scores for assignment 3 ranged from 0 to 5, with every correct subtraction receiving a score of 1. Finally, the subjects were required to recall the 10 words that had been previously remembered in assignment 1. The subjects were given a score of 1 for each correct word, with the possible scores for assignment 4 ranging from 0 to 10. Therefore, the range of the overall global cognitive score was 0–27. A higher score on the global cognitive function assessment indicated a greater level of functional cognition. A cross-sectional study in China indicated that the prevalence of mild cognitive impairment was 20% in Chinese aged 60 and over [20]. In this study, we determined that the first quintile of the cognitive score, which equates to a cutoff of 7, represents poor cognitive function.

#### 2.2.2. Dietary Consumption

The CHNS used the 24 h dietary recall over 3 consecutive days to collect individual dietary information. The dietary intake of each individual was collected over 3 days by a well-trained investigator to conduct a 24 h dietary recall. In addition, at the start and end of the survey period of 3 consecutive days, investigators chose the household food weighing method to weigh and document foods and condiments from the home inventory, as well as household food waste and household foods obtained from markets or garden harvests. Methods of dietary collection have been described in detail elsewhere [14,21]. The dietary collection method has been previously validated [22]. In this study, dietary patterns were identified using individual-level dietary information, except for oils and salts, which were determined using the household weighing method. Daily average food intake per person was analyzed using the Chinese Food Composition Table (FCT) and expressed in grams (g).

#### 2.2.3. Dietary Pattern and Score Derivation

Based on the Mediterranean diet developed by Trichopoulou et al., the AMED score was created [23]. In this study, we used the AMED score modified by Fung et al., which was based on scientific evidence linking foods, nutrients, and chronic disease risk [24]. The AMED score was defined by adding up scores for 9 different components, comprising vegetables, legumes, nuts, fruits, whole grains, fish, the ratio of monounsaturated fatty acid (MUFA) to saturated fatty acid (SFA), red, and processed meats and alcohol. Each component was assigned a score of 1 if its intake met or exceeded the median level of healthy constituents, such as vegetables, legumes, nuts, fruits, whole grains, and fish, and the ratio of MUFA to SFA, while remaining below the median intake of unhealthy constituents, such as red and processed meats. Participants were assigned a score of 1 to indicate their alcohol consumption, as defined by a range of ≥5 g and ≤15 g per day for females and ≥10 g and ≤25 g per day for males.

The DASH score was defined by 8 different components, comprising vegetables, fruits, legumes and nuts, whole grains, low-fat dairy products, sugar-sweetened beverages and juices, sodium, and red and processed meats [25]. On the basis of their intake ranking, all participants were divided into fifths for each component. The fifth rankings were determined by assessing the scores of each of the five essential components of a healthy diet, including vegetables, fruits, legumes and nuts, whole grains, and low-fat dairy products. As an illustration, the first fifth received a score of 1, whereas the fifth received a score of 5. However, low intake is preferred for sugar-sweetened beverages and juices, sodium, and red and processed meats. Hence for each of these 3 components, the lowest fifth received a score of 5, while the highest fifth received a score of 1. The component scores were summed to give individuals a DASH score that ranged from 8 to 40. In this study, low-fat dairy was not collected in the CHNS, so the DASH score was calculated using dairy rather than low-fat dairy [26]. The DASH score has been applied to prior studies about the risk of chronic diseases such as cardiovascular disease, etc. 

The CHFP score consisted of 10 food groups: (1) grains, potatoes and beans; (2) vegetables; (3) fruits; (4) meat; (5) eggs; (6) seafood; (7) dairy products; (8) nuts and soybean products; (9) cooking oil; (10) salt [27]. We used the CHFP score established by Qin et al. based on a study of the CHNS [28]. Subjects were assigned a score of 1 for each food group if they consumed sufficient food to meet the recommended intake. A score of 0.5 was given if subjects consumed food from a specific food group in the range of 50% more or less than the recommended amount. Conversely, if the food consumed by a subject did not fall within the recommended range or the range of 50% more or less than the recommended amount, a score of 0 was assigned. As a result, the CHFP score ranged from 0 to 10.

#### 2.2.4. Covariates

Information on demographics, lifestyle risk factors (e.g., smoking status and energy intake), and health status (e.g., history of diabetes, stroke, and hypertension) was collected by trained investigators using structured questionnaires. The variables for demographics included age, sex (male, female), residential region (city, rural), geographic location (north, south), education (low: illiterate or primary school, medium: junior middle school, and high: high middle school or technical secondary school or above), per capita annual family income, and marital status (never married, married and divorced, widowed or separated). The smoking status was categorized as never, ever, and current. Diabetes and stroke were classified as binary variables, with individuals self-reporting their presence or absence.

Hypertension was operationally defined as meeting the criteria of having a systolic blood pressure ≥ 140 mmHg and/or a diastolic blood pressure ≥ 90 mmHg, being under medication for hypertension, or self-reporting a diagnosis of hypertension. Following a period of rest lasting no less than 5 min in a tranquil setting, the investigators proceeded to assess the blood pressure (BP) of the seated participant by conducting three measurements on the right arm utilizing mercury sphygmomanometers that were routinely calibrated. Before the measurement, subjects were instructed to refrain from smoking, drinking caffeinated beverages or alcohol, and taking exercise for at least 30 min. Additionally, participants were instructed to remove their shoes and wear lightweight clothing during the process of measuring their height and weight, with precision to the nearest 0.1 cm and 0.1 kg, respectively [29]. Body mass index (BMI) was calculated using the measured height (m) and weight (kg). Based on the Guidelines for the Prevention and Control of Overweight and Obesity in Chinese Adults, BMI values < 18.5, between 18.5 and 23.9, and > 23.9 kg/m^2^ were utilized to denote underweight, normal weight, and overweight and obesity, respectively [30].

### 2.3. Statistical Analysis

Each dietary pattern score was divided into tertiles and tied values were classified as the lower tertile. The overall assessment of general characteristics was conducted, taking into account the cognitive function status, and subjected to statistical analysis. The chi-squared test was employed to compare differences across groups for categorical variables, while Student’s *t*-test, the Mann–Whitney *U* test, ANOVA, or the Kruskal–Wallis test were utilized for continuous variables to assess differences across groups.

Binary logistic regression models were used to calculate the odds ratio (OR) of poor cognitive function for each dietary pattern score, based on diet tertile. The odds ratio (OR) for poor cognitive function was determined by categorizing dietary pattern scores into tertiles. The lowest tertile (Tertile 1), which was considered the least healthy tertile, was used as the reference group in both the crude and adjusted models. Model 1 incorporated demographic adjustments, model 2 additionally accounted for lifestyle risk factors, and model 3 further incorporated adjustments for medical history and BMI. In the subgroup analyses, the logistic regression model incorporated a product term to calculate the multiplicative interaction between adherence to each dietary pattern and covariates, such as sex, residential region, geographic location, education, hypertension, and BMI. In the sensitivity analyses, we excluded those with histories of diabetes and stroke, respectively. The analyses were performed using Python and the IBM Statistical Package for the Social Sciences, version 25.0 (IBM-SPSS Inc., Chicago, IL, USA). Results were considered statistically significant when *p* < 0.05 (two-tailed).

## 3. Results

### 3.1. Descriptive Results

A total of 3353 subjects attending cognitive screening tests were included after excluding those with missing dietary information. The characteristics of the subjects are presented in Table 1. The mean ± SD age was 65.5 ± 8.2 years, 47.6% of subjects were male, 36.7% lived in the city, 41.1% lived in the north, and 58.9% lived in the south. Individuals exhibiting poor cognition (global cognition scores < 7) demonstrated a higher likelihood of being female, residing in rural and southern regions, possessing lower income and education levels, consuming less energy, being unmarried, and having hypertension and stroke.

Supplementary Table S1 presents the characteristics of the dietary pattern score tertiles, wherein individuals adhering to a healthier diet exhibited a higher propensity for marriage, urban residency, northern geographical location, and elevated income and education levels.

### 3.2. Association between Dietary Patterns and Poor Cognition

The results of the logistic regression models are presented in Table 2. A negative association between high adherence to the AMED, DASH, and CHFP diets and poor cognition was found. Model 3 holds significant importance as it represents the most comprehensive and adequately adjusted model, accounting for all factors that exhibited significant differences in the univariate analysis.

Among the crude model of the AMED diet score, there was a notable decrease in ORs (95% CIs) for poor cognition across tertiles of 1 (reference), 0.679 (0.551, 0.836), and 0.364 (0.292, 0.455), respectively. In the adjusted models, the association was attenuated, and the significant relationship was no longer observed in the second tertile. However, the AMED diet score was statistically significant for the third tertile of the adjusted models, the ORs (95% CIs) of models 1, 2, and 3 were 0.572 (0.446, 0.734), 0.605 (0.468,0.782), and 0.594 (0.458, 0.771), respectively. The test for linear trends had statistical significance in all models.

The ORs (95% CIs) for the consecutive categories of the DASH diet score were 1 (reference), 0.930 (0.742, 1.164), and 0.652 (0.504, 0.843) in model 3. None of the associations for the crude and three adjusted models exhibited statistical significance in the second tertile. For all models, the test for linear trends was statistically significant.

Likewise, a statistically significant association was observed between the healthiest tertile of the CHFP diet score and a decreased risk of global cognition scores < 7 in all models. Comparatively, when compared to the least unhealthy tertile, the ORs (95% CIs) of the healthiest tertile for model 1, model 2, and model 3 were 0.634 (0.444, 0.906), 0.590 (0.412, 0.845), and 0.599 (0.417, 0.861), respectively. Similarly to the AMED diet score and DASH diet score, the test for linear trends was also statistically significant for all models.

### 3.3. Subgroup Analysis of the Association between Dietary Patterns and Poor Cognition

As shown in Table 3, a significant interaction (*p* for interaction = 0.045) between high adherence to the AMED and residential regions with poor cognition was found. The negative association of high adherence to the AMED on poor cognition was statistically significant among those in the city (OR = 0.413; 95%CI = 0.259, 0.658; *p* < 0.001), but not in rural areas (OR = 0.730; 95%CI = 0.532, 1.002; *p* = 0.067). There was no significant variation observed in the association between high adherence to the AMED and poor cognition across different strata, including sex, geographic location, education level, hypertension status, and BMI categories.

There were interactions between residential regions (*p* for interaction = 0.003), education (*p* for interaction = 0.048), and high adherence to the DASH diet concerning poor cognition (Table 4). The inverse association between high adherence to the DASH diet and poor cognition was statistically significant among those in the city (OR = 0.362; 95%CI = 0.218, 0.601; *p* < 0.001), but no such association was observed among those in rural areas (OR = 0.857; 95%CI = 0.632, 1.163; *p* = 0.354). The inverse association was stronger among those with a low level of education (OR = 0.742; 95%CI = 0.567, 0.971; *p* = 0.036) compared to those with a high level of education (OR = 0.036; 95%CI = 0.004, 0.340; *p* = 0.001). There was no interaction with sex, geographic location, hypertension, and BMI.

High adherence to the CHFP had no interactions with sex, geographic location, education, hypertension, and BMI concerning the association with poor cognition. Similarly to the AMED and DASH diets, a notable distinction in residential regions was observed within the association (city: OR = 0.228; 95%CI = 0.104, 0.500; *p* < 0.001 and rural: OR = 0.992; 95%CI = 0.645, 1.525; *p* = 0.812). Specifically, the association was only observed among those in the city but not those in rural areas (*p* for interaction < 0.001). The specific results of the subgroup analysis are shown in Table 5.

### 3.4. Sensitivity Analysis

After excluding those with a history of diabetes, the results remained consistent (Supplementary Table S2). Similarly, the results did not change significantly after excluding those with a history of stroke (Supplementary Table S3).

## 4. Discussion

This study investigated the association between adherence to three different dietary patterns and cognitive function. We found that high adherence to the AMED, DASH, and CHFP diets was positively associated with a reduced risk of poor cognition in Chinese middle-aged and elderly adults. In addition, high adherence to the AMED, DASH, and CHFP diets exhibited remarkable interactions with residential regions. To the best of our knowledge, this is the first study to report associations between cognitive function and adherence to the CHFP among middle-aged and elderly adults.

The AMED diet exhibited the strongest association with reduced risk of poor cognition among all dietary patterns, whereas the CHFP diet displayed the second strongest association, immediately following the AMED diet. Lastly, the DASH diet demonstrated a comparatively weaker association with a decreased risk of poor cognition. Our findings showed that following the AMED was more beneficial for improving cognition in the Chinese middle-aged and elderly populations, followed by the CHFP developed based on the CDG. Furthermore, it is evident from both the crude and adjusted models that all dietary patterns displayed a distinct linear trend, indicating a consistent negative association between dietary pattern scores and the prevalence of poor cognition, and their associations followed a dose–response pattern.

Most previous studies have shown that the Mediterranean diet is associated with improved cognitive function [31,32,33,34]. A cross-sectional study in Spain showed that the Mediterranean diet was positively related to cognitive function [35]. A subsequent cross-sectional study conducted on a sample of 970 individuals aged 60 years and older within the Australian population revealed that individuals diagnosed with mild cognitive impairment (MCI) and Alzheimer’s disease (AD) exhibited poorer adherence to the Mediterranean diet compared to their healthy counterparts [36]. Similarly, longitudinal studies indicated that high adherence to the Mediterranean diet is associated with a reduced risk of cognitive decline [37,38,39]. For example, a prospective cohort study published in JAMA showed that higher adherence to the Mediterranean diet was associated with slower cognitive decline as measured by the Mini-Mental State Examination (MMSE) [40]. A study in 2021 conducted on 1046 seniors with a follow-up period of about 3 years also showed that a higher level of adherence to the Mediterranean diet was associated with a lower risk of cognitive decline and incident dementia [41]. These findings are consistent with our analysis. However, Brolsma et al. reported that higher Mediterranean diet scores are not linked to better cognitive scores [42]. A randomized controlled trial in France showed no association between the Mediterranean diet and cognitive performance when subjects were evaluated 13 years later [11]. A large Harvard study of older women observed no association between the Mediterranean diet and cognitive decline [43]. The incongruous outcomes observed in the relationship between AMED diets and cognitive decline may be attributed to variations in the duration of follow-up, characteristics of the study population, criteria employed for diet scoring, and potential inaccuracies in dietary data collection, among other factors. To fully comprehend the relationship, more research on the association between cognitive performance and adherence to the AMED is required. 

Similarly to the Mediterranean diet, results for the association between the DASH diet and cognition are inconsistent. A study of 923 subjects found that higher adherence to the DASH diet was associated with lower AD rates [44]. Wengreen et al. conducted a prospective cohort study on elderly individuals, which revealed that greater adherence to the DASH diet exhibited a positive association with enhanced cognitive function [45]. However, other cohort studies also revealed that there is no significant association between increased adherence to the DASH diet and cognitive function [46,47]. The reason for the conflicting results between the DASH diet and cognitive function may be due to the different follow-up times, physiological differences in the study population, and other reasons, and further studies on their relationships with cognition are required.

Some research concentrated on exploring the association of the CHFP diet with cardiovascular disease [48,49,50]. Only Zhu et al. explored the relationship between the CHFP diet and memory and decision making [51]. As far as we know, ours is the first study to explore the CHFP diet concerning cognitive function. Our analysis revealed a significant inverse association between adherence to the CHFP diet and poor cognition, suggesting that a high-quality CHFP diet may play a crucial role in enhancing cognitive function. Future research should include longitudinal studies to further investigate and validate this association.

The mechanisms relating dietary patterns to cognitive function still need to be elucidated. As a combination of various food types and quantities in daily dietary intake, the dietary pattern takes into account the interaction, synergy, and accumulation of potential effects between food and nutrients, which might be mainly responsible for the significant enhancement in cognitive function [8]. Specifically, the three dietary patterns in our study all contain fruits, vegetables, legumes, and grains, with abundant bioactive compounds present in them, including carotenoids, unsaturated fatty acids, antioxidant vitamins, and polyphenols [52]. Previous research has provided evidence for the efficacy of these nutrients in diminishing the amyloid burden, enhancing neurogenesis and neuritogenesis, fostering synaptic plasticity, and supporting neuronal survival via the mitigation of oxidative stress and neuroinflammation, thereby resulting in cognitive improvement [53,54,55]. 

In addition, evidence in recent years has suggested that the gut microbiota influences cognitive function via the gut–brain axis [56,57,58,59]. Dietary patterns can selectively promote the growth of gut bacteria, like *Bifidobacteria* and *Prevotella*, and some fiber-degrading bacteria, such as *Bacteroides cellulosilyticus*, *Eubacterium eligens*, and *Faecalibacterium prausnitzii*, resulting in lowered levels of metabolic endotoxemia such as trimethylamine oxide and LPS and increased levels of short-chain fatty acids (SCFAs) [60,61,62,63,64]. A recent study of 612 participants from five different countries revealed that adhering to the Mediterranean diet selectively increased the abundance of anti-inflammatory and SCFA-producing bacteria, diminished inflammatory markers, and increased levels of branched fatty acids and SCFAs, which contributed to better cognitive function in the elderly [65].

The intriguing aspect lies in the interaction between the residential region and the level of adherence to the AMED, DASH, and CHFP diets in relation to cognitive function. The inverse association between high adherence to the AMED, DASH, and CHFP diets and poor cognition was only observed among those who lived in the city. This could be related to the notably higher prevalence of cognitive impairment in rural areas compared with that in urban ones in China. The prevalence of dementia is 6.05% in rural areas and 4.40% in urban areas according to a population-based cross-sectional survey across China [66]. Our study also showed that the prevalence of poor cognition was 22.4% in rural areas and 14.8% in urban areas. The rural region may have more significant factors impacting the prevalence of dementia other than dietary patterns, such as lack of education, socialization, cultural and recreational activities, and medical resources, and in particular education might be an important reason for the differences [67,68,69]. Studies showed that individuals in rural areas have clearly lower levels of education than those in urban areas [66,70]. Therefore, dietary improvements may not achieve cognitive improvements for individuals in rural areas across China. In addition, there was an interaction between adherence to the DASH diet and education, and the negative association between high adherence to the DASH diet and poor cognition was only observed among those with high and low levels of education. This may be because those with high and low levels of education are more likely to follow dietary patterns. Additionally, the association between adherence to the DASH diet and cognition may differ by educational attainment. Notably, the inverse relationship between high adherence to the DASH diet and cognitive performance was solely evident among individuals with both high and low levels of education. This observation suggests that individuals with higher and lower education levels are more inclined to adopt specific dietary patterns, potentially explaining the observed association.

It is important to acknowledge the strengths and limitations inherent in this study. Instead of investigating only one dietary pattern, as in most studies, our study analyzed and compared the relationship between several dietary patterns and cognitive functions. Our research findings contribute to filling the existing gap in the literature regarding the association between the CHFP diet and cognitive function in the middle-aged and elderly population. This gap arises from the lack of prior exploration of the relationship between the CHFP diet score and cognitive function. Thirdly, the utilization of a substantial sample encompassing diverse provinces facilitated the generalization of our findings to a wider population. In addition, we were able to control a variety of potential confounders. Regarding limitations, it is important to note that the cognitive function test items employed by the CHNS currently lack a universally accepted cutoff value. However, it is possible to establish a suitable cut-off value by drawing upon the findings of numerous prior high-quality studies. Another limitation is that since no data were collected specifically on low-fat dairy products in the CHNS, the total dairy replaced low-fat dairy consumption. In addition, the objects of this study were limited to Chinese middle-aged and elderly people aged 55 and above; therefore, the findings might not be generalizable to younger and more diverse populations.

## 5. Conclusions

In summary, our findings indicate that maintaining a high adherence to the AMED, DASH, and CHFP diets is significantly linked to a reduced risk of poor cognition, particularly among urban populations. Importantly, our study is the first to establish a connection between the CHFP diet and cognitive function in the elderly. Of course, future longitudinal investigations should be carried out to validate this link. Dietary guidelines for the prevention of cognitive decline should consider the need for additional research and utilization of these dietary patterns. It is also important to explore the underlying mechanisms that link dietary patterns with cognition in greater depth.

## Figures and Tables

**Table 1:** Sample characteristics of subjects overall and by cognitive state.

	Global Cognition Scores < 7 (*N* = 658)	Global Cognition Scores ≥ 7 (*N* = 2695)	Overall (*N* = 3353)	*p*
Age (years), mean ± SD	71.3 ± 9.1	64.0 ± 7.3	65.5 ± 8.2	<0.001
Sex				<0.001
Male	209 (31.8)	1388 (51.5)	1597 (47.6)	
Female	449 (68.2)	1307 (48.5)	1756 (52.4)	
Residential region				<0.001
City	182 (27.7)	1048 (38.9)	1230 (36.7)	
Rural	476 (72.3)	1647 (61.1)	2123 (63.3)	
Geographic location				<0.001
North	215 (32.7)	1164 (43.2)	1379 (41.1)	
South	443 (67.3)	1531 (56.8)	1974 (58.9)	
Education				<0.001
Low	613 (93.2)	786 (29.2)	2347 (70.0)	
Medium	27 (4.1)	907 (33.7)	516 (15.4)	
High	18 (2.7)	1002 (37.2)	490 (14.6)	
Income (CNY), median (IQR)	3617.53 (1999.5, 7472.0)	6703.7 (3105.1, 12,906.0)	5835.3 (2711.7, 11,627.6)	<0.001
Marital status				<0.001
Never married	8 (1.2)	18 (0.7)	26 (0.8)	
Married	386 (58.7)	2246 (83.3)	2632 (78.5)	
Divorced, widowed, or separated	264 (40.1)	431 (16.0)	695 (20.7)	
Smoking status				<0.001
Never	509 (77.4)	1723 (63.9)	2232 (66.6)	
Ever	43 (6.5)	221 (8.2)	264 (7.9)	
Current	106 (16.1)	751 (27.9)	857 (25.6)	
Energy intake				<0.001
Low	329 (50.5)	788 (29.2)	1117 (33.3)	
Medium	193 (29.3)	926 (34.4)	1119 (33.4)	
High	136 (20.7)	981 (36.4)	1117 (33.3)	
Hypertension				0.039
No	368 (55.9)	1626 (60.3)	1994 (59.5)	
Yes	290 (44.1)	1069 (39.7)	1359 (40.5)	
Diabetes				0.426
No	630 (95.7)	2598 (96.4)	3228 (96.3)	
Yes	28 (4.3)	97 (3.6)	125 (3.7)	
Stroke				<0.001
No	618 (93.9)	2644 (98.1)	3262 (97.3)	
Yes	40 (6.1)	51 (1.9)	91 (2.7)	
BMI				<0.001
Underweight	73 (11.1)	172 (6.4)	245 (7.3)	
Normal weight	409 (62.2)	1414 (52.5)	1823 (54.4)	
Overweight and obesity	176 (26.7)	1109 (41.2)	1285 (38.3)	

Data are presented as n (%) unless otherwise shown. SD, standard deviation; IQR, interquartile range.

**Table 2:** ORs (95% CIs) for global cognitive score < 7 by tertiles of dietary patterns among Chinese middle-aged and elderly adults.

	*N* (%)	Prevalence of Poor Cognition	Crude	Model 1	Model 2	Model 3
AMED						
Tertile 1	1442 (43.0)	26.0	Ref	Ref	Ref	Ref
Tertile 2	836 (24.9)	19.3	**0.679 (0.551, 0.836)**	0.834 (0.660, 1.054)	0.862 (0.680, 1.092)	0.862 (0.678, 1.095)
Tertile 3	1075 (32.1)	11.1	**0.364 (0.292, 0.455)**	**0.572 (0.446, 0.734)**	**0.605 (0.468, 0.782)**	**0.594 (0.458, 0.771)**
*p* for trend			**<0.001**	**<0.001**	**<0.001**	**<0.001**
DASH						
Tertile 1	1313 (39.2)	23.2	Ref	Ref	Ref	Ref
Tertile 2	1050 (31.3)	20.8	0.866 (0.711, 1.054)	0.914 (0.732, 1.142)	0.926 (0.741, 1.158)	0.930 (0.742, 1.164)
Tertile 3	990 (29.5)	13.6	**0.522 (0.418, 0.652)**	**0.643 (0.500, 0.827)**	**0.664 (0.515, 0.855)**	**0.652 (0.504, 0.843)**
*p* for trend			**<0.001**	**0.001**	**0.002**	**0.001**
CHFP						
Tertile 1	1163 (34.7)	20.9	Ref	Ref	Ref	Ref
Tertile 2	1755 (52.3)	20.6	0.984 (0.820, 1.181)	1.020 (0.829, 1.254)	0.982 (0.797, 1.210)	1.004 (0.813, 1.239)
Tertile 3	435 (13.0)	12.2	**0.525 (0.381, 0.724)**	**0.634 (0.444, 0.906)**	**0.590 (0.412, 0.845)**	**0.599 (0.417, 0.861)**
*p* for trend			**<0.001**	**0.024**	**0.007**	**0.011**

Data are ORs (95% CI) unless otherwise shown. Ref, reference; AMED, Alternate Mediterranean Diet; DASH, Dietary Approaches to Stop Hypertension; CHFP, Chinese Food Pagoda. Crude model was the unadjusted model. Model 1 adjusted for age, sex, residential region, geographic location, education, income, and marital status. Model 2 further adjusted for smoking status and energy intake. Model 3 further adjusted for hypertension, stroke, and BMI. CI estimated using modified binary logistic regression model. Boldface type indicates statistical significance at the *p* < 0.05 level.

**Table 3:** Subgroup analysis of the association between tertiles of AMED and global cognitive score < 7.

		AMED		*p* for Trend	*p* for Interaction
	Tertile 1	Tertile 2	Tertile 3		
Sex					0.743
Male	Ref	0.998 (0.671, 1.485)	**0.581 (0.370, 0.912)**	**0.031**	
Female	Ref	0.798 (0.589, 1.081)	**0.599 (0.434, 0.828)**	**0.002**	
Residential region					**0.045**
City	Ref	0.701 (0.448, 1.095)	**0.413 (0.259, 0.658)**	**<0.001**	
Rural	Ref	0.957 (0.717, 1.276)	0.730 (0.532, 1.002)	0.067	
Geographic location					0.287
North	Ref	0.913 (0.603, 1.382)	0.793 (0.515, 1.223)	0.297	
South	Ref	0.839 (0.623, 1.129)	**0.501 (0.358, 0.699)**	**<0.001**	
Education					0.974
Low	Ref	0.872 (0.679, 1.119)	**0.603 (0.458, 0.795)**	**<0.001**	
Medium	Ref	1.002 (0.331, 3.030)	0.435 (0.127, 1.495)	0.218	
High	Ref	0.453 (0.106, 1.933)	0.392 (0.111, 1.385)	0.144	
Hypertension					0.983
No	Ref	0.839 (0.610, 1.155)	**0.577 (0.402, 0.828)**	**0.003**	
Yes	Ref	0.890 (0.618, 1.283)	**0.613 (0.417, 0.900)**	**0.015**	
BMI					0.530
Underweight	Ref	1.083 (0.445, 2.640)	0.929 (0.362, 2.384)	0.935	
Normal weight	Ref	0.784 (0.573, 1.073)	**0.548 (0.386, 0.779)**	**<0.001**	
Overweight and obesity	Ref	0.993 (0.650, 1.517)	**0.581 (0.368, 0.917)**	**0.026**	

Data are OR (95% CI) unless otherwise shown. AMED, Alternate Mediterranean Diet; Ref, reference. Logistic models adjusted for age, sex, residential region, geographic location, education, income, marital status, smoking status, energy intake, hypertension, stroke, and BMI. Each stratification variable was not adjusted in the corresponding model. Boldface type indicates statistical significance at the *p* < 0.05 level.

**Table 4:** Subgroup analysis of the association between tertiles of DASH and global cognitive score < 7.

		DASH		*p* for Trend	*p* for Interaction
	Tertile 1	Tertile 2	Tertile 3		
Sex					0.593
Male	Ref	0.783 (0.535, 1.146)	**0.626 (0.401, 0.979)**	**0.035**	
Female	Ref	1.023 (0.771, 1.357)	**0.662 (0.482, 0.908)**	**0.014**	
Residential region					**0.003**
City	Ref	0.781 (0.512, 1.190)	**0.362 (0.218, 0.601)**	**<0.001**	
Rural	Ref	1.035 (0.790, 1.356)	0.857 (0.632, 1.163)	0.354	
Geographic location					0.325
North	Ref	1.135 (0.749, 1.720)	0.832 (0.541, 1.279)	0.351	
South	Ref	0.877 (0.668, 1.152)	**0.584 (0.418, 0.817)**	**0.002**	
Education					**0.048**
Low	Ref	0.992 (0.784, 1.257)	**0.742 (0.567, 0.971)**	**0.036**	
Medium	Ref	0.360 (0.114, 1.137)	0.277 (0.077, 0.999)	**0.034**	
High	Ref	0.593 (0.182, 1.932)	**0.036 (0.004, 0.340)**	**0.001**	
Hypertension					0.958
No	Ref	0.960 (0.715, 1.289)	**0.631 (0.441, 0.903)**	**0.016**	
Yes	Ref	0.884 (0.620, 1.261)	**0.651 (0.446, 0.948)**	**0.026**	
BMI					0.103
Underweight	Ref	1.607 (0.751, 3.439)	1.060 (0.443, 2.540)	0.806	
Normal weight	Ref	0.742 (0.550, 1.000)	**0.597 (0.425, 0.839)**	**0.002**	
Overweight and obesity	Ref	1.143 (0.761, 1.719)	0.635 (0.399, 1.010)	0.059	

Data are OR (95% CI) unless otherwise shown. DASH, Dietary Approaches to Stop Hypertension; Ref, reference. Logistic models adjusted for age, sex, residential region, geographic location, education, income, marital status, smoking status, energy intake, hypertension, stroke, and BMI. Each stratification variable was not adjusted in the corresponding model. Boldface type indicates statistical significance at the *p* < 0.05 level.

**Table 5:** Subgroup analysis of the association between tertiles of CHFP and global cognitive score < 7.

		CHFP		*p* for Trend	*p* for Interaction
	Tertile 1	Tertile 2	Tertile 3		
Sex					0.214
Male	Ref	0.793 (0.555, 1.133)	0.580 (0.324, 1.037)	0.056	
Female	Ref	1.155 (0.888, 1.503)	**0.588 (0.369, 0.939)**	0.070	
Residential region					**<0.001**
City	Ref	1.297 (0.855, 1.967)	**0.228 (0.104, 0.500)**	**<0.001**	
Rural	Ref	0.907 (0.707, 1.164)	0.992 (0.645, 1.525)	0.812	
Geographic location					0.342
North	Ref	0.919 (0.636, 1.329)	0.765 (0.422, 1.385)	0.372	
South	Ref	1.058 (0.817, 1.372)	**0.522 (0.327, 0.832)**	**0.015**	
Education					0.790
Low	Ref	0.981 (0.787, 1.223)	**0.580 (0.393, 0.857)**	**0.012**	
Medium	Ref	1.014 (0.377, 2.727)	0.561 (0.136, 2.305)	0.416	
High	Ref	2.468 (0.610, 9.991)	1.166 (0.189, 7.201)	0.947	
Hypertension					0.840
No	Ref	1.001 (0.757, 1.324)	**0.515 (0.307, 0.864)**	**0.023**	
Yes	Ref	0.993 (0.718, 1.374)	0.666 (0.395, 1.122)	0.149	
BMI					0.329
Underweight	Ref	1.145 (0.579, 2.264)	0.480 (0.106, 2.180)	0.539	
Normal weight	Ref	0.917 (0.697, 1.208)	0.737 (0.463, 1.172)	0.195	
Overweight and obesity	Ref	1.169 (0.789, 1.732)	**0.448 (0.227, 0.886)**	**0.031**	

Data are OR (95% CI) unless otherwise shown. CHFP, Chinese Food Pagoda; Ref, reference. Logistic models adjusted for age, sex, residential region, geographic location, education, income, marital status, smoking status, energy intake, hypertension, stroke, and BMI. Each stratification variable was not adjusted in the corresponding model. Boldface type indicates statistical significance at the *p* < 0.05 level.

## Data Availability

The datasets generated and analyzed during this investigation may be found in the CHNS repository, which can be found at https://www.cpc.unc.edu/projects/china accessed on 9 January 2022.

## Supplementary Materials

The following supporting information can be downloaded at https://www.mdpi.com/article/10.3390/nu15183974/s1, Table S1: Sample characteristics of subjects by tertile of dietary pattern score; Table S2: ORs (95% CIs) for global cognitive score < 7 by tertiles of dietary patterns after excluding people with a history of diabetes; Table S3: ORs (95% CIs) for global cognitive score < 7 by tertiles of dietary patterns after excluding people with a history of stroke.

## References

[1] Scheltens P., De Strooper B., Kivipelto M., Holstege H., Chételat G., Teunissen C.E., Cummings J., van der Flier W.M. (2021). Alzheimer’s disease. Lancet.

[2] WHO (2022). Dementia.

[3] (2023). 2023 Alzheimer’s disease facts and figures. Alzheimers Dement..

[4] Jia J., Wei C., Chen S., Li F., Tang Y., Qin W., Zhao L., Jin H., Xu H., Wang F. (2018). The cost of Alzheimer’s disease in China and re-estimation of costs worldwide. Alzheimers Dement..

[5] Xu M., Ke P., Wang C., Xia W., Meng X., Di H., Gan Y., He Y., Tian Q., Jiang H. (2022). Association of food groups with the risk of cognitive impairment in Chinese older adults. J. Affect. Disord..

[6] Wei K., Yang J., Lin S., Mei Y., An N., Cao X., Jiang L., Liu C., Li C. (2022). Dietary Habits Modify the Association of Physical Exercise with Cognitive Impairment in Community-Dwelling Older Adults. J. Clin. Med..

[7] Solfrizzi V., Custodero C., Lozupone M., Imbimbo B.P., Valiani V., Agosti P., Schilardi A., D’Introno A., La Montagna M., Calvani M. (2017). Relationships of Dietary Patterns, Foods, and Micro- and Macronutrients with Alzheimer’s Disease and Late-Life Cognitive Disorders: A Systematic Review. J. Alzheimers Dis..

[8] Chu C.Q., Yu L.L., Qi G.Y., Mi Y.S., Wu W.Q., Lee Y.K., Zhai Q.X., Tian F.W., Chen W. (2022). Can dietary patterns prevent cognitive impairment and reduce Alzheimer’s disease risk: Exploring the underlying mechanisms of effects. Neurosci. Biobehav. Rev..

[9] Valls-Pedret C., Sala-Vila A., Serra-Mir M., Corella D., de la Torre R., Martínez-González M.Á., Martínez-Lapiscina E.H., Fitó M., Pérez-Heras A., Salas-Salvadó J. (2015). Mediterranean Diet and Age-Related Cognitive Decline: A Randomized Clinical Trial. JAMA Intern. Med..

[10] Nagpal R., Neth B.J., Wang S., Craft S., Yadav H. (2019). Modified Mediterranean-ketogenic diet modulates gut microbiome and short-chain fatty acids in association with Alzheimer’s disease markers in subjects with mild cognitive impairment. EBioMedicine.

[11] Kesse-Guyot E., Andreeva V.A., Lassale C., Ferry M., Jeandel C., Hercberg S., Galan P. (2013). Mediterranean diet and cognitive function: A French study. Am. J. Clin. Nutr..

[12] Smith P.J., Blumenthal J.A., Babyak M.A., Craighead L., Welsh-Bohmer K.A., Browndyke J.N., Strauman T.A., Sherwood A. (2010). Effects of the dietary approaches to stop hypertension diet, exercise, and caloric restriction on neurocognition in overweight adults with high blood pressure. Hypertension.

[13] Lee E., Kady V., Han E., Montan K., Normuminova M., Rovito M.J. (2022). Healthy Eating and Mortality among Breast Cancer Survivors: A Systematic Review and Meta-Analysis of Cohort Studies. Int. J. Environ. Res. Public Health.

[14] Zhai F.Y., Du S.F., Wang Z.H., Zhang J.G., Du W.W., Popkin B.M. (2014). Dynamics of the Chinese diet and the role of urbanicity, 1991–2011. Obes. Rev..

[15] Popkin B.M., Du S., Zhai F., Zhang B. (2010). Cohort Profile: The China Health and Nutrition Survey--monitoring and understanding socio-economic and health change in China, 1989–2011. Int. J. Epidemiol..

[16] Duff K., Tometich D., Dennett K. (2015). The Modified Telephone Interview for Cognitive Status is More Predictive of Memory Abilities Than the Mini-Mental State Examination. J. Geriatr. Psychiatry Neurol..

[17] Plassman B.L., Welsh K.A., Helms M., Brandt J., Page W.F., Breitner J.C. (1995). Intelligence and education as predictors of cognitive state in late life: A 50-year follow-up. Neurology.

[18] Potter G.G., Helms M.J., Plassman B.L. (2008). Associations of job demands and intelligence with cognitive performance among men in late life. Neurology.

[19] Yao Y., Wang K., Xiang H. (2022). Association between cognitive function and ambient particulate matters in middle-aged and elderly Chinese adults: Evidence from the China Health and Retirement Longitudinal Study (CHARLS). Sci. Total Environ..

[20] Ding D., Zhao Q., Guo Q., Meng H., Wang B., Luo J., Mortimer J.A., Borenstein A.R., Hong Z. (2015). Prevalence of mild cognitive impairment in an urban community in China: A cross-sectional analysis of the Shanghai Aging Study. Alzheimers Dement..

[21] Zhao R., Zhao L., Gao X., Yang F., Yang Y., Fang H., Ju L., Xu X., Guo Q., Li S. (2022). Geographic Variations in Dietary Patterns and Their Associations with Overweight/Obesity and Hypertension in China: Findings from China Nutrition and Health Surveillance (2015–2017). Nutrients.

[22] Yu D., Zhao L., Zhao W. (2020). Status and trends in consumption of grains and dietary fiber among Chinese adults (1982–2015). Nutr. Rev..

[23] Trichopoulou A., Costacou T., Bamia C., Trichopoulos D. (2003). Adherence to a Mediterranean diet and survival in a Greek population. N. Engl. J. Med..

[24] Fung T.T., Hu F.B., McCullough M.L., Newby P.K., Willett W.C., Holmes M.D. (2006). Diet quality is associated with the risk of estrogen receptor-negative breast cancer in postmenopausal women. J. Nutr..

[25] Appel L.J., Moore T.J., Obarzanek E., Vollmer W.M., Svetkey L.P., Sacks F.M., Bray G.A., Vogt T.M., Cutler J.A., Windhauser M.M. (1997). A clinical trial of the effects of dietary patterns on blood pressure. DASH Collaborative Research Group. N. Engl. J. Med..

[26] Chen G.C., Koh W.P., Neelakantan N., Yuan J.M., Qin L.Q., van Dam R.M. (2018). Diet Quality Indices and Risk of Type 2 Diabetes Mellitus: The Singapore Chinese Health Study. Am. J. Epidemiol..

[27] Nguyen S., Li H., Yu D., Gao J., Gao Y., Tran H., Xiang Y.B., Zheng W., Shu X.O. (2020). Adherence to dietary recommendations and colorectal cancer risk: Results from two prospective cohort studies. Int. J. Epidemiol..

[28] Qin B., Adair L.S., Plassman B.L., Batis C., Edwards L.J., Popkin B.M., Mendez M.A. (2015). Dietary Patterns and Cognitive Decline Among Chinese Older Adults. Epidemiology.

[29] Huang Q., Jiang H., Zhang B., Wang H., Jia X., Huang F., Wang L., Wang Z. (2019). Threshold-Effect Association of Dietary Cholesterol Intake with Dyslipidemia in Chinese Adults: Results from the China Health and Nutrition Survey in 2015. Nutrients.

[30] Gao M., Wei Y.X., Lyu J., Yu C.Q., Guo Y., Bian Z., Pei P., Du H.D., Chen J.S., Chen Z.M. (2019). The cut-off points of body mass index and waist circumference for predicting metabolic risk factors in Chinese adults. Zhonghua Liu Xing Bing Xue Za Zhi.

[31] Limongi F., Siviero P., Bozanic A., Noale M., Veronese N., Maggi S. (2020). The Effect of Adherence to the Mediterranean Diet on Late-Life Cognitive Disorders: A Systematic Review. J. Am. Med. Dir. Assoc..

[32] Coelho-Júnior H.J., Trichopoulou A., Panza F. (2021). Cross-sectional and longitudinal associations between adherence to Mediterranean diet with physical performance and cognitive function in older adults: A systematic review and meta-analysis. Ageing Res. Rev..

[33] Fu J., Tan L.J., Lee J.E., Shin S. (2022). Association between the mediterranean diet and cognitive health among healthy adults: A systematic review and meta-analysis. Front. Nutr..

[34] Singh B., Parsaik A.K., Mielke M.M., Erwin P.J., Knopman D.S., Petersen R.C., Roberts R.O. (2014). Association of mediterranean diet with mild cognitive impairment and Alzheimer’s disease: A systematic review and meta-analysis. J. Alzheimers Dis..

[35] Hernández-Galiot A., Goñi I. (2017). Adherence to the Mediterranean diet pattern, cognitive status and depressive symptoms in an elderly non-institutionalized population. Nutr. Hosp..

[36] Gardener S., Gu Y., Rainey-Smith S.R., Keogh J.B., Clifton P.M., Mathieson S.L., Taddei K., Mondal A., Ward V.K., Scarmeas N. (2012). Adherence to a Mediterranean diet and Alzheimer’s disease risk in an Australian population. Transl. Psychiatry.

[37] Carlos S., De La Fuente-Arrillaga C., Bes-Rastrollo M., Razquin C., Rico-Campà A., Martínez-González M.A., Ruiz-Canela M. (2018). Mediterranean Diet and Health Outcomes in the SUN Cohort. Nutrients.

[38] Shannon O.M., Stephan B., Granic A., Lentjes M., Hayat S., Mulligan A., Brayne C., Khaw K.T., Bundy R., Aldred S. (2019). Mediterranean diet adherence and cognitive function in older UK adults: The European Prospective Investigation into Cancer and Nutrition-Norfolk (EPIC-Norfolk) Study. Am. J. Clin. Nutr..

[39] Bhushan A., Fondell E., Ascherio A., Yuan C., Grodstein F., Willett W. (2018). Adherence to Mediterranean diet and subjective cognitive function in men. Eur. J. Epidemiol..

[40] Féart C., Samieri C., Rondeau V., Amieva H., Portet F., Dartigues J.F., Scarmeas N., Barberger-Gateau P. (2009). Adherence to a Mediterranean diet, cognitive decline, and risk of dementia. JAMA.

[41] Charisis S., Ntanasi E., Yannakoulia M., Anastasiou C.A., Kosmidis M.H., Dardiotis E., Hadjigeorgiou G., Sakka P., Scarmeas N. (2021). Mediterranean diet and risk for dementia and cognitive decline in a Mediterranean population. J. Am. Geriatr. Soc..

[42] Brouwer-Brolsma E.M., Benati A., van de Wiel A., van Lee L., de Vries J., Feskens E., van de Rest O. (2018). Higher Mediterranean Diet scores are not cross-sectionally associated with better cognitive scores in 20- to 70-year-old Dutch adults: The NQplus study. Nutr. Res..

[43] Samieri C., Grodstein F., Rosner B.A., Kang J.H., Cook N.R., Manson J.E., Buring J.E., Willett W.C., Okereke O.I. (2013). Mediterranean diet and cognitive function in older age. Epidemiology.

[44] Morris M.C., Tangney C.C., Wang Y., Sacks F.M., Bennett D.A., Aggarwal N.T. (2015). MIND diet associated with reduced incidence of Alzheimer’s disease. Alzheimers Dement..

[45] Wengreen H., Munger R.G., Cutler A., Quach A., Bowles A., Corcoran C., Tschanz J.T., Norton M.C., Welsh-Bohmer K.A. (2013). Prospective study of Dietary Approaches to Stop Hypertension- and Mediterranean-style dietary patterns and age-related cognitive change: The Cache County Study on Memory, Health and Aging. Am. J. Clin. Nutr..

[46] Shakersain B., Rizzuto D., Larsson S.C., Faxén-Irving G., Fratiglioni L., Xu W.L. (2018). The Nordic Prudent Diet Reduces Risk of Cognitive Decline in the Swedish Older Adults: A Population-Based Cohort Study. Nutrients.

[47] Haring B., Wu C., Mossavar-Rahmani Y., Snetselaar L., Brunner R., Wallace R.B., Neuhouser M.L., Wassertheil-Smoller S. (2016). No Association between Dietary Patterns and Risk for Cognitive Decline in Older Women with 9-Year Follow-Up: Data from the Women’s Health Initiative Memory Study. J. Acad. Nutr. Diet..

[48] Wang F., Cai H., Gu K., Shi L., Yu D., Zhang M., Zheng W., Zheng Y., Bao P., Shu X.O. (2020). Adherence to Dietary Recommendations among Long-Term Breast Cancer Survivors and Cancer Outcome Associations. Cancer Epidemiol. Biomark. Prev..

[49] Zhou J., Leepromrath S., Zhou D. (2022). Dietary diversity indices versus dietary guideline-based indices and their associations with non-communicable diseases, overweight and energy intake: Evidence from China. Public Health Nutr..

[50] Yu D., Zhang X., Xiang Y.B., Yang G., Li H., Gao Y.T., Zheng W., Shu X.O. (2014). Adherence to dietary guidelines and mortality: A report from prospective cohort studies of 134,000 Chinese adults in urban Shanghai. Am. J. Clin. Nutr..

[51] Zhu J., Xiang Y.B., Cai H., Li H., Gao Y.T., Zheng W., Shu X.O. (2018). A Prospective Investigation of Dietary Intake and Functional Impairments Among the Elderly. Am. J. Epidemiol..

[52] Scarmeas N., Anastasiou C.A., Yannakoulia M. (2018). Nutrition and prevention of cognitive impairment. Lancet Neurol..

[53] Mohammadzadeh H.N., Saedisomeolia A., Abdolahi M., Shayeganrad A., Taheri S.G., Hassanzadeh R.B., Muench G. (2017). Molecular Anti-inflammatory Mechanisms of Retinoids and Carotenoids in Alzheimer’s Disease: A Review of Current Evidence. J. Mol. Neurosci..

[54] Lakey-Beitia J., Kumar D.J., Hegde M.L., Rao K.S. (2019). Carotenoids as Novel Therapeutic Molecules Against Neurodegenerative Disorders: Chemistry and Molecular Docking Analysis. Int. J. Mol. Sci..

[55] Zeqaj I., Piffero R., Calzaducca E., Pirisi M., Bellan M. (2023). The Potential Role of Vitamin D Supplementation In Cognitive Impairment Prevention. CNS & Neurological Disorders—Drug Targets.

[56] Cryan J.F., O’Riordan K.J., Sandhu K., Peterson V., Dinan T.G. (2020). The gut microbiome in neurological disorders. Lancet Neurol..

[57] Fang P., Kazmi S.A., Jameson K.G., Hsiao E.Y. (2020). The Microbiome as a Modifier of Neurodegenerative Disease Risk. Cell Host Microbe.

[58] Agirman G., Yu K.B., Hsiao E.Y. (2021). Signaling inflammation across the gut-brain axis. Science.

[59] Chakrabarti A., Geurts L., Hoyles L., Iozzo P., Kraneveld A.D., La Fata G., Miani M., Patterson E., Pot B., Shortt C. (2022). The microbiota-gut-brain axis: Pathways to better brain health. Perspectives on what we know, what we need to investigate and how to put knowledge into practice. Cell. Mol. Life Sci..

[60] Wang D.D., Nguyen L.H., Li Y., Yan Y., Ma W., Rinott E., Ivey K.L., Shai I., Willett W.C., Hu F.B. (2021). The gut microbiome modulates the protective association between a Mediterranean diet and cardiometabolic disease risk. Nat. Med..

[61] Meslier V., Laiola M., Roager H.M., De Filippis F., Roume H., Quinquis B., Giacco R., Mennella I., Ferracane R., Pons N. (2020). Mediterranean diet intervention in overweight and obese subjects lowers plasma cholesterol and causes changes in the gut microbiome and metabolome independently of energy intake. Gut.

[62] Bailey M.A., Holscher H.D. (2018). Microbiome-Mediated Effects of the Mediterranean Diet on Inflammation. Adv. Nutr..

[63] Bourdeau-Julien I., Castonguay-Paradis S., Rochefort G., Perron J., Lamarche B., Flamand N., Di Marzo V., Veilleux A., Raymond F. (2023). The diet rapidly and differentially affects the gut microbiota and host lipid mediators in a healthy population. Microbiome.

[64] Merra G., Noce A., Marrone G., Cintoni M., Tarsitano M.G., Capacci A., De Lorenzo A. (2020). Influence of Mediterranean Diet on Human Gut Microbiota. Nutrients.

[65] Ghosh T.S., Rampelli S., Jeffery I.B., Santoro A., Neto M., Capri M., Giampieri E., Jennings A., Candela M., Turroni S. (2020). Mediterranean diet intervention alters the gut microbiome in older people reducing frailty and improving health status: The NU-AGE 1-year dietary intervention across five European countries. Gut.

[66] Jia J., Wang F., Wei C., Zhou A., Jia X., Li F., Tang M., Chu L., Zhou Y., Zhou C. (2014). The prevalence of dementia in urban and rural areas of China. Alzheimer’s Dement. J. Alzheimer’s Assoc..

[67] Cheng C., Yang C.Y., Inder K., Wai-Chi Chan S. (2020). Urban-rural differences in mental health among Chinese patients with multiple chronic conditions. Int. J. Ment. Health Nurs..

[68] Lu S., Li Y., Gao H., Zhang Y. (2022). Difference in bypass for inpatient care and its determinants between rural and urban residents in China. Int. J. Equity Health.

[69] Li Y., Zhang X., Sang H., Niu X., Liu T., Liu W., Li J. (2019). Urban-rural differences in risk factors for ischemic stroke in northern China. Medicine.

[70] Cao L.J., Yan X.K. (2016). Urban-Rural Differences in Returns to education: A Phased Study. Fudan Educ. Forum.

