# Supplementary material for: Higher Adherence to the AMED, DASH, and CHFP Dietary Patterns Is Associated with Better Cognition among Chinese Middle-Aged and Elderly Adults
Source: Nutrients. 2023 Sep 14;15(18):3974. doi: 10.3390/nu15183974 (PMC10535050; doi:10.3390/nu15183974)
Supplement: Supplementary file 1 [file nutrients-15-03974-s001.zip › nutrients-2532588-Supplementary materials.pdf]

Supplementary Table S1. Sample characteristics of subjects by tertile of dietary pattern score.

	AMED				DASH				CHFP			
	**Tertile 1 (1442)**	**Tertile 2 (836)**	**Tertile 3 (1075)**	***P***	**Tertile 1 (1313)**	**Tertile 2 (1050)**	**Tertile 3 (990)**	***P***	**Tertile 1 (1163)**	**Tertile 2 (1755)**	**Tertile 3 (435)**	***P***
Age (years), mean ± SD	66.2 ± 8.6	64.9 ± 8.1	64.9 ± 7.7	<0.001	65.6 ± 8.4	65.3 ± 8.2	65.5 ± 8.0	0.723	65.2 ± 8.0	65.6 ± 8.4	65.6 ± 7.9	0.558
Sex				0.289				0.533				0.991
Male	668 (46.3)	397 (47.5)	532 (49.5)		636 (48.4)	504 (48.0)	457 (46.2)		555 (47.7)	834 (47.5)	208 (47.8)	
Female	774 (53.7)	439 (52.5)	543 (50.5)		677 (51.6)	546 (52.0)	533 (53.8)		608 (52.3)	921 (52.5)	227 (52.2)	
Residential region				<0.001				<0.001				<0.001
City	392 (27.2)	292 (34.9)	546 (50.8)		426 (32.4)	334 (31.8)	470 (47.5)		319 (27.4)	678 (38.6)	233 (53.6)	
Rural	1050 (72.8)	544 (65.1)	529 (49.2)		887 (67.6)	716 (68.2)	520 (52.5)		844 (72.6)	1077 (61.4)	202 (46.4)	
Geographic location				<0.001				<0.001				0.013
North	511 (35.4)	370 (44.3)	498 (46.3)		431 (32.8)	439 (41.8)	509 (51.4)		466 (40.1)	706 (40.2)	207 (47.6)	
South	931 (64.6)	466 (55.7)	577 (53.7)		882 (67.2)	611 (58.2)	481 (48.6)		697 (59.9)	1049 (59.8)	228 (52.4)	
Education				<0.001				<0.001				0.001
Low	1112 (77.1)	607 (72.6)	628 (58.4)		964 (73.4)	766 (73.0)	617 (62.3)		845 (72.7)	1230 (70.1)	272 (62.5)	
Medium	191 (13.2)	128 (15.3)	197 (18.3)		198 (15.1)	159 (15.1)	159 (16.1)		177 (15.2)	258 (14.7)	81 (18.6)	
High	139 (9.6)	101 (12.1)	250 (23.3)		151 (11.5)	125 (11.9)	214 (21.6)		141 (12.1)	267 (15.2)	82 (18.9)	
Income (¥), median (IQR)	4770.6 (2391.7, 8926.2)	5273.2 (2371.4, 10738.2)	8564.10 (4346.2, 16025.6)	<0.001	5286.1 (2500.0, 10290.0)	5243.8 (2549.0, 10224.9)	8086.9 (3511.7, 15985.8)	<0.001	5104.0 (2440.0, 9750.0)	5907.7 (2782.6, 11940.3)	8416.7 (4312.5, 13949.6)	<0.001
Marital status				<0.001				0.011				0.018

Never married	15 (1.0)	8 (1.0)	3 (0.3)		16 (1.2)	6 (0.6)	4 (0.4)		12 (1.0)	11 (0.6)	3 (0.7)	
Married	1066 (73.9)	655 (78.3)	911 (84.7)		997 (75.9)	832 (79.2)	803 (81.1)		902 (77.6)	1363 (77.7)	367 (84.4)	
Divorced, widowed or separated	361 (25.0)	173 (20.7)	161 (15.0)		300 (22.8)	212 (20.2)	183 (18.5)		249 (21.4)	381 (21.7)	65 (14.9)	
Smoking status				0.066				0.005				0.287
Never	964 (66.9)	564 (67.5)	704 (65.5)		892 (67.9)	663 (63.1)	677 (68.4)		757 (65.1)	1176 (67.0)	299 (68.7)	
Ever	101 (7.0)	57 (6.8)	106 (9.9)		93 (7.1)	81 (7.7)	90 (9.1)		92 (7.9)	132 (7.5)	40 (9.2)	
Current	377 (26.1)	215 (25.7)	265 (24.7)		328 (25.0)	306 (29.1)	223 (22.5)		314 (27.0)	447 (25.5)	96 (22.1)	
Energy intake				<0.001				<0.001				<0.001
Low	646 (44.8)	251 (30.0)	220 (20.5)		486 (37.0)	365 (34.8)	266 (26.9)		304 (26.1)	629 (35.8)	184 (42.3)	
Medium	461 (32.0)	279 (33.4)	379 (35.3)		414 (31.5)	360 (34.3)	345 (34.8)		389 (33.4)	570 (32.5)	160 (36.8)	
High	335 (23.2)	306 (36.6)	476 (44.3)		413 (31.5)	325 (31.0)	379 (38.3)		470 (40.4)	556 (31.7)	91 (20.9)	
Hypertension				<0.001				0.003				0.176
No	912 (63.2)	491 (58.7)	591 (55.0)		805 (61.3)	645 (61.4)	544 (54.9)		696 (59.8)	1057 (60.2)	241 (55.4)	
Yes	530 (36.8)	345 (41.3)	484 (45.0)		508 (38.7)	405 (38.6)	446 (45.1)		467 (40.2)	698 (39.8)	194 (44.6)	
Diabetes				<0.001				<0.001				0.002
No	1403 (97.3)	810 (96.9)	1015 (94.4)		1284 (97.8)	1016 (96.8)	928 (93.7)		1129 (97.1)	1693 (96.5)	406 (93.3)	
Yes	39 (2.7)	26 (3.1)	60 (5.6)		29 (2.2)	34 (3.2)	62 (6.3)		34 (2.9)	62 (3.5)	29 (6.7)	
Stroke				0.418				0.465				0.117
No	1409 (97.7)	811 (97.0)	1042 (96.9)		1283 (97.7)	1018 (97.0)	961 (97.1)		1131 (97.2)	1714 (97.7)	417 (95.9)	
Yes	33 (2.3)	25 (3.0)	33 (3.1)		30 (2.3)	32 (3.0)	29 (2.9)		32 (2.8)	41 (2.3)	18 (4.1)	
BMI				<0.001				<0.001				<0.001
Underweight	144 (10.0)	49 (5.9)	52 (4.8)		103 (7.8)	80 (7.6)	62 (6.3)		97 (8.3)	131 (7.5)	17 (3.9)	
Normal	867 (60.1)	440 (52.6)	516 (48.0)		769 (58.6)	561 (53.4)	493 (49.8)		669 (57.5)	950 (54.1)	204 (46.9)	

weight												
Overweight and obesity	431 (29.9)	347 (41.5)	507 (47.2)		441 (33.6)	409 (39.0)	435 (43.9)		397 (34.1)	674 (38.4)	214 (49.2)	

Data are presented as *n* (%) unless otherwise shown. AMED, Alternate Mediterranean Diet; DASH, Dietary Approaches to Stop Hypertension; CHFP, Chinese Food Pagoda; SD, standard deviation; IQR, interquartile range.

Supplementary Table S2. ORs (95% CIs) for global cognitive score <7 by tertiles of dietary patterns after excluding people with a history of diabetes.

	**Crude**	**Model 1**	**Model 2**	**Model 3**
AMED				
Tertile 1	Ref	Ref	Ref	Ref
Tertile 2	**0.667 (0.539, 0.825)**	0.839 (0.660, 1.065)	0.866 (0.680, 1.102)	0.866 (0.678, 1.105)
Tertile 3	**0.362 (0.288, 0.456)**	**0.575 (0.445, 0.743)**	**0.605 (0.465, 0.787)**	**0.596 (0.456, 0.779)**
p for trend	**<0.001**	**<0.001**	**<0.001**	**<0.001**
DASH				
Tertile 1	Ref	Ref	Ref	Ref
Tertile 2	0.823 (0.673, 1.006)	0.863 (0.688,1.083)	0.876 (0.697,1.100)	0.872 (0.693, 1.098)
Tertile 3	**0.527 (0.420, 0.661)**	**0.650 (0.503,0.840)**	**0.670 (0.517,0.868)**	**0.654 (0.503, 0.850)**
p for trend	**<0.001**	**0.001**	**0.003**	**0.002**
CHFP				
Tertile 1	Ref	Ref	Ref	Ref
Tertile 2	0.970 (0.805, 1.169)	0.995 (0.806, 1.229)	0.960 (0.776, 1.188)	0.984 (0.794, 1.221)
Tertile 3	**0.547 (0.394, 0.758)**	**0.652 (0.453, 0.939)**	**0.610 (0.422, 0.880)**	**0.614 (0.423, 0.890)**
p for trend	**0.001**	**0.035**	**0.013**	**0.017**

Data are OR (95% CI) unless otherwise shown. Ref, reference; AMED, Alternate Mediterranean Diet; DASH, Dietary Approaches to Stop Hypertension; CHFP, Chinese Food Pagoda. Crude model was the unadjusted model. Model 1 adjusted for age, gender, residential region, geographic location, education, income and marital status. Model 2 further adjusted for smoking status and energy intake. Model 3 further adjusted for hypertension, stroke and BMI. CI estimated through modified binary logistic regression model. Boldface type means statistical significance at the p <0.05 level.

Supplementary Table S3. ORs (95% CIs) for global cognitive score <7 by tertiles of dietary patterns after excluding people with a history of stroke.

	**Crude**	**Model 1**	**Model 2**	**Model 3**
AMED				
Tertile 1	Ref	Ref	Ref	Ref
Tertile 2	**0.658 (0.531, 0.816)**	0.813 (0.639, 1.035)	0.835 (0.655, 1.065)	0.848 (0.664, 1.083)
Tertile 3	**0.345 (0.274, 0.435)**	**0.559 (0.431, 0.724)**	**0.583 (0.447, 0.761)**	**0.595 (0.456, 0.778)**
p for trend	**<0.001**	**<0.001**	**<0.001**	**<0.001**
DASH				
Tertile 1	Ref	Ref	Ref	Ref
Tertile 2	0.836 (0.684, 1.023)	0.896 (0.713, 1.126)	0.905 (0.720, 1.138)	0.922 (0.732, 1.160)
Tertile 3	**0.496 (0.394, 0.624)**	**0.629 (0.485, 0.816)**	**0.647 (0.498, 0.841)**	**0.651 (0.500, 0.847)**
p for trend	**<0.001**	**0.001**	**0.001**	**0.002**
CHFP				
Tertile 1	Ref	Ref	Ref	Ref
Tertile 2	1.028 (0.852, 1.240)	1.090 (0.881, 1.348)	1.054 (0.850, 1.306)	1.072 (0.864, 1.330)
Tertile 3	**0.514 (0.367, 0.720)**	**0.615 (0.423, 0.895)**	**0.577 (0.395, 0.842)**	**0.598 (0.409, 0.874)**
p for trend	**<0.001**	**0.031**	**0.011**	**0.020**

Data are OR (95% CI) unless otherwise shown. Ref, reference; AMED, Alternate Mediterranean Diet; DASH, Dietary Approaches to Stop Hypertension; CHFP, Chinese Food Pagoda. Crude model was the unadjusted model. Model 1 adjusted for age, gender, residential region, geographic location, education, income and marital status. Model 2 further adjusted for smoking status and energy intake. Model 3 further adjusted for hypertension, stroke and BMI. CI estimated through modified binary logistic regression model. Boldface type means statistical significance at the $p < 0.05$ level.
